# The role of the exercise physiology laboratory in disease management: pulmonary arterial hypertension

**DOI:** 10.36416/1806-3756/e20240240

**Published:** 2024-09-16

**Authors:** Eloara V M Ferreira, Julina S Lucena, Rudolf K F Oliveira

**Affiliations:** 1. Divisão de Pneumologia, Escola Paulista de Medicina, Universidade Federal de São Paulo, São Paulo (SP) Brasil.

Pulmonary arterial hypertension (PAH) is a rare disease associated with exercise intolerance due to right ventricular dysfunction. Risk stratification in PAH is essential in defining the prognosis, and cardiopulmonary exercise testing (CPET) provides valuable information about the severity of the disease, with direct implications for clinical management.

## OVERVIEW

**Figure 1:**
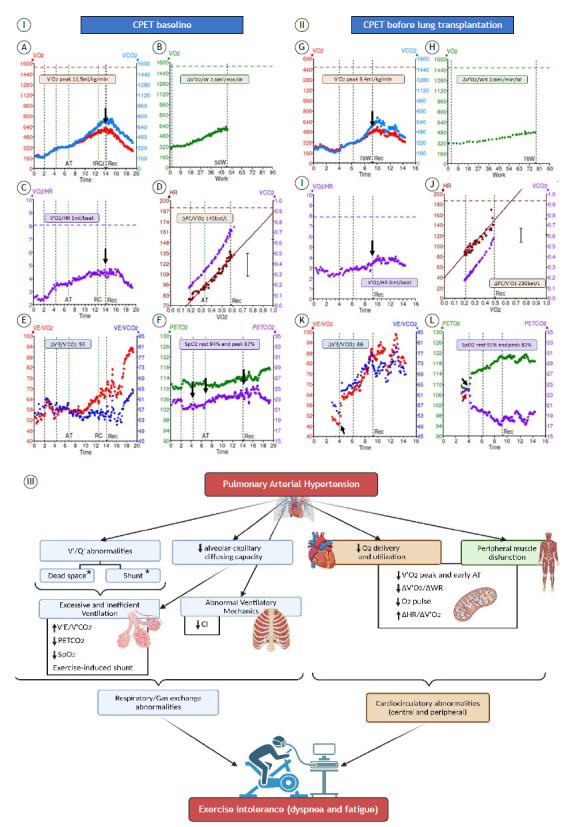
A 33-year-old woman diagnosed with pulmonary arterial hypertension (PAH) 11 years prior, using triple therapy, and on the waiting list for lung transplantation. Panel I (A-F) - Cardiopulmonary exercise testing (CPET) at baseline showed the following: markedly reduced aerobic capacity and signs of cardiocirculatory limitation analyzed by a reduced peak V^•^ O_2_ (V^•^ O_2peak_ ) with slow recovery (A); ∆V^•^ O_2_/∆WR with a plateau at the end of exercise (B); early anaerobic threshold (AT), and a steeper response of the ∆HR/∆VO_2peak_, corresponding to a tachycardic pattern (D); and O_2_ pulse (V^•^ O_2_/HR) with an early plateau (C) ; excessive ventilatory responses (increased ∆V^•^ E/∆V^•^ CO_2_ ) suggestive of ventilation-perfusion (V/Q ) mismatch (E); and reduced PETCO_2_ associated with exercise-induced desaturation (F). The Borg scale scores were 4 for dyspnea, 7 for fatigue, and 1.21 for the respiratory exchange ratio (RER) peak. Panel II (G-L) - CPET performed at one year before lung transplantation showed worsening of all of the responses and add-on signs of right-to-left shunt marked by an abrupt decrease in PETCO_2_ (red arrow in L) simultaneous to an abrupt increase in V^•^ E/V^•^ CO_2_ and V^•^ E/V^•^ O_2_ (black arrow in K ) and PETO_2_ (black arrow in L) with a rise in RER and worsening of exercise-induced desaturation. The Borg scale scores were 9 for dyspnea, 9 for fatigue, and 1.19 for the RER peak. Panel III - Summary of the main CPET findings in patients with PAH. *It is necessary to collect blood samples to perform gas exchange analysis and calculate the ratio of dead space to tidal volume.

A 33-year-old woman, diagnosed with PAH 11 years prior, currently using triple combination therapy, and on the waiting list for lung transplantation, underwent CPET at the time of diagnosis (Figure 1-Panel I) and again at one year before lung transplantation (Figure 1-Panel II). Although both examinations revealed reduced aerobic capacity, signs of cardiocirculatory limitation, and excessive ventilatory responses with gas exchange disturbance, the latter also showed indirect signs of a right-to-left shunt. In patients with PAH, CPET is a valuable tool in assessing prognosis.^1^ The primary variable is the peak V^•^ O_2_ (V^•^ O_2peak_), which is related to oxygen delivery and consumption. If the oxygen delivery is low, the first response of the muscle is to increase the oxygen consumption. However, in PAH, this mechanism could fail with a high dependence on non-oxidative pathways, low energy production, and increased exercise intolerance. In addition, patients with PAH have low type I muscle fiber density, which impairs peripheral oxygen use and limits muscle strength. During high metabolic demand, patients with PAH have a lower V^•^ O_2peak_, early anaerobic threshold (AT), and low aerobic efficiency with a low V^•^ O_2_-work rate (∆V^•^ O_2_/∆WR) or a plateau response.^2^ Ultimately, PAH leads to a reduction in stroke volume that requires a compensatory increase in HR to maintain cardiac output, which leads to a steeper ∆HR/∆V^•^ O_2_ response, a reduced V^•^ O_2peak_, and O_2_ pulse (V^•^ O_2_/HR) with a curve plateau before or after the AT.^3^ In the PAH lungs, low perfusion with adequate alveolar ventilation results in ventilation-perfusion mismatch and gas exchange disturbance during exercise, increasing ventilatory demand. This augmented response is also related to lactic acid accumulation, signaling peripheral chemoreceptors and increasing the feedback for ventilation. In this context, a high minute ventilation to carbon dioxide production ratio (V^•^ E/V^•^ CO_2_) and lower end-tidal carbon dioxide partial pressure (PETCO_2_) values have been reported, suggesting signs of ventilatory inefficiency in PAH.^3^ In addition, PAH patients usually experience decreased SpO_2_ from rest to peak exercise, also related to exercise-induced shunt (EIS).^2^ Right-to-left shunting during exercise is attributable to an abnormally high pulmonary vascular resistance to right atrial pressure exceeding left atrial pressure, forcing systemic venous blood through a patent foramen ovale directly into the systemic arterial circulation.^4^ An abrupt and sustained decrease in PETCO_2_ associated with a simultaneous and sustained increase in PETO_2_, in the ventilatory equivalents of oxygen and carbon dioxide (V^•^ E/V^•^ O_2_ and V^•^ E/V^•^ CO_2_), and in the respiratory exchange ratio (RER), together with a decline in SpO_2_, are findings present in patients with EIS.^5^ The persistence or development of an EIS strongly predicts death or transplantation regardless of the hemodynamics and all other CPET variables.^4^ In the European Respiratory Society guideline, the peak V^•^ O_2peak_ and the V^•^ E/V^•^ CO_2_ slope have established cutoff values for prognosis assessment. However, other analyses are not considered in the guideline but have provided pathophysiological evidence of an impact on disease severity, such analyses including the assessment of a right-to-left shunt.^5^

## CLINICAL MESSAGE

In PAH risk stratification, CPET can play an important role, having the advantage of being noninvasive. This tool might facilitate the decision-making process and clinical management in patients with PAH.
